# Supplementary material for: Effects of Citrulline or Watermelon Supplementation on Body Composition: A Systematic Review and Dose–Response Meta-Analysis
Source: Nutrients. 2025 Sep 30;17(19):3126. doi: 10.3390/nu17193126 (PMC12526429; doi:10.3390/nu17193126)
Supplement: Supplementary file 1 [file nutrients-17-03126-s001.zip › nutrients-3863969-supplementary.pdf]

Supporting Information

Table S1. Risk of bias assessment for included RCTs in the meta-analysis

**References**	Bias arising from the randomization process (Allocation bias)	Bias due to deviations from the intended interventions (Performance bias)	Bias due to missing outcome data (Attrition bias)	Bias in the measurement of the Outcome (Detection bias)	results (Reporting bias)	The overall risk of bias
Aghabeigi et al.2020	U	U	L	U	U	H
Azizi et al.2021	L	L	L	L	L	L
Bouillanne et al.2019	L	L	L	L	L	L
Buckinx et al.2018	L	L	L	L	L	L
Buckinx et al.2019	L	L	L	L	L	L
Buckinx et al.2020	L	L	L	L	L	L
Burgos et al.2022	L	L	L	L	L	L
Abbaszadeh et al.2021	L	L	L	L	L	L
Darabi et al.2019	L	L	L	L	L	L
Ellis et al.2021	L	L	L	L	L	L
Figueroa et al.2015	H	U	L	L	U	H
Hoseinzadeh et al.2021	L	L	L	L	L	L
Hwang et al.2018	L	L	L	L	L	L
Kang et al.2022	L	L	L	L	L	L
Marcangeli et al.2022	L	L	L	L	L	L
Moradi et al.2019	U	U	L	L	U	H
Rafee et al.2020	U	U	L	L	U	H
Shanely et al.2020	H	U	L	L	U	H
Wong et al.2016	U	H	L	L	U	H
Wong et al.2016	U	H	L	L	U	H
Youssef et al.2023	L	L	L	L	L	L

Abbreviations: L, low risk of bias; H, high risk of bias; U, unclear risk of bias

Table S2. GRADE assessment

Outcomes	Risk of bias	Inconsistency	Indirectness	Imprecision	Publication Bias	Quality of evidence
Body weight	No serious limitation	No serious limitation	No serious limitation	Serious limitation[3]	No serious limitation	⊕⊕⊕⊖ Moderate
BMI	No serious limitation	No serious limitation	No serious limitation	No serious limitation	No serious limitation	⊕⊕⊕⊕ High
FM	No serious limitation	No serious limitation	No serious limitation	Serious limitation[3]	Serious limitation[4]	⊕⊕⊖⊖ Low
BFP	Serious limitation[1]	No serious limitation	No serious limitation	No serious limitation	No serious limitation	⊕⊕⊕⊖ Moderate
FFM	No serious limitation	Serious limitation[2]	No serious limitation	Serious limitation[3]	No serious limitation	⊕⊕⊖⊖ Low
WC	No serious limitation	No serious limitation	No serious limitation	Serious limitation[3]	No serious limitation	⊕⊕⊕⊖ Moderate

Abbreviations: BMI, body mass index; FFM, fat-free mass; FM, fat mass; BFP, body fat percentage; WC, waist circumference

[1] It was downgraded due to the high risk of bias.
[2] It was downgraded due to the presence of significant heterogeneity (I^2>50%).
[3] It was downgraded because the confidence interval for the effect size range was > 2.
[4] There was publication bias based on Egger's and Begg's tests.

A) Body weight

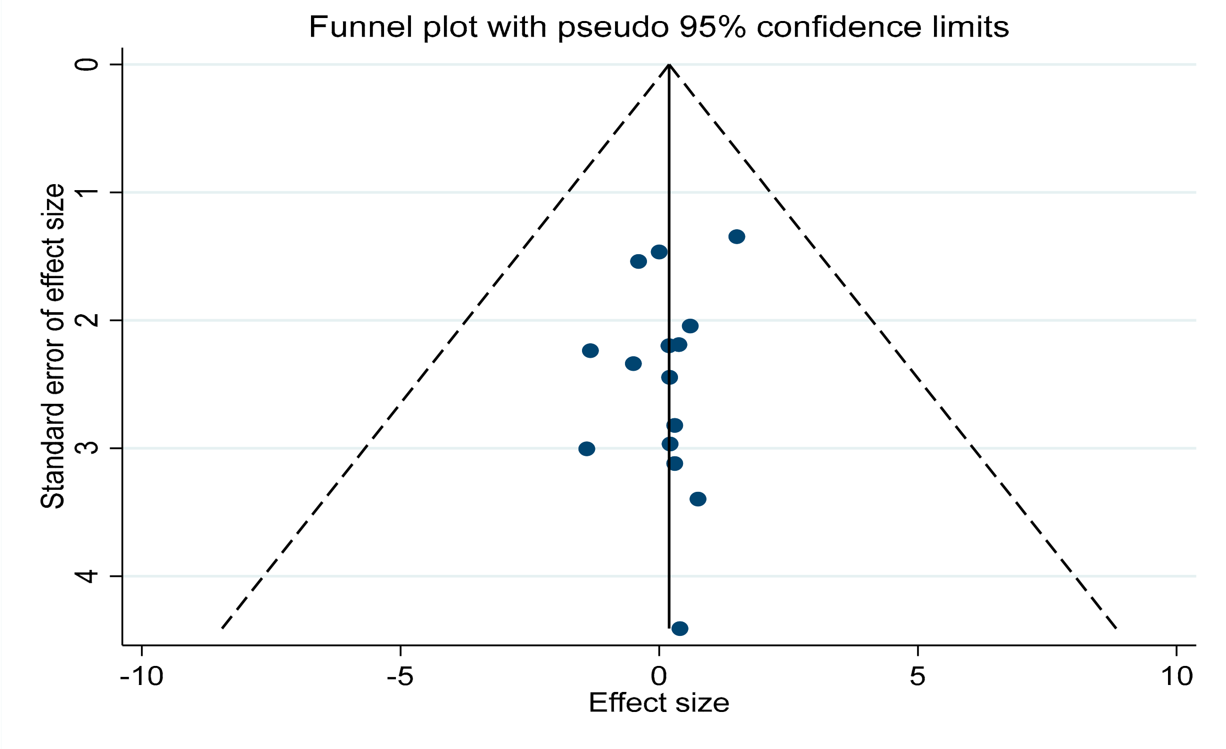

B) Body mass index (BMI)

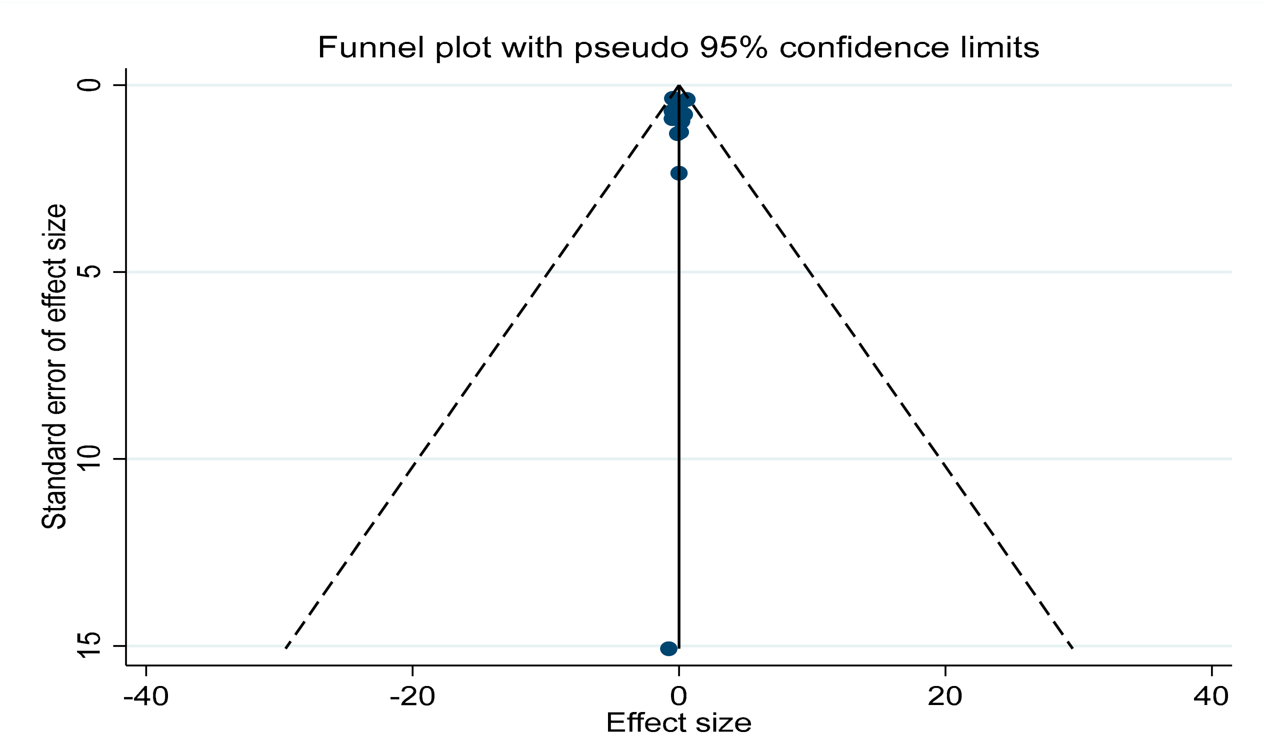

C) Fat mass (FM)

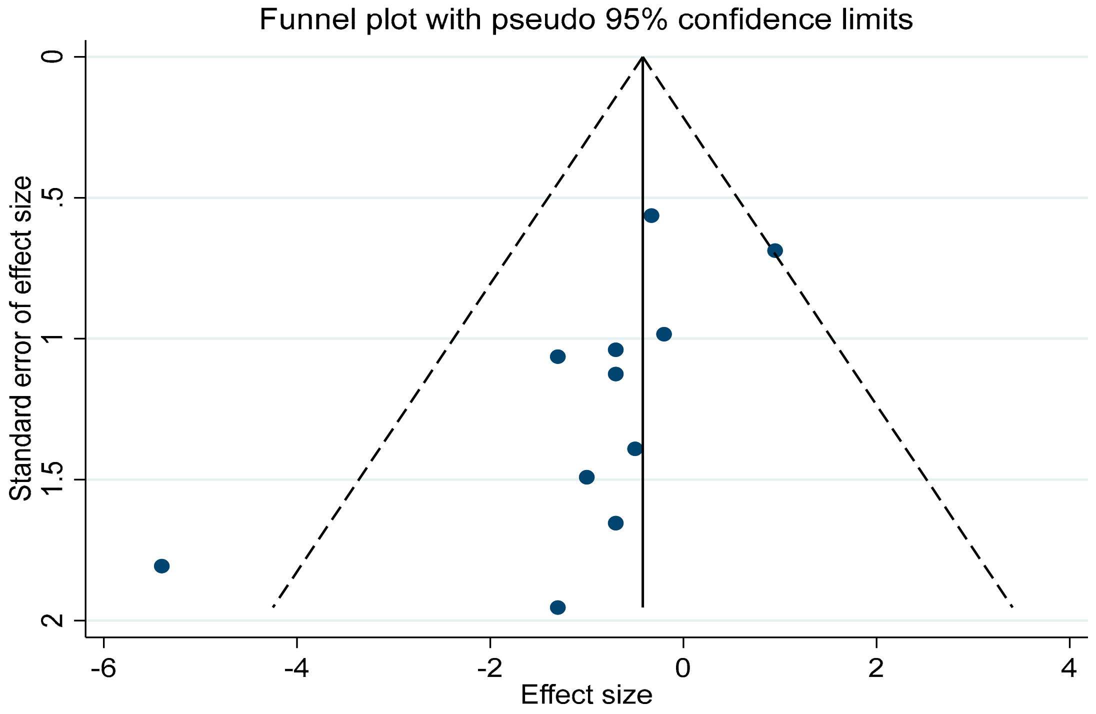

D) Body fat percentage (BFP)

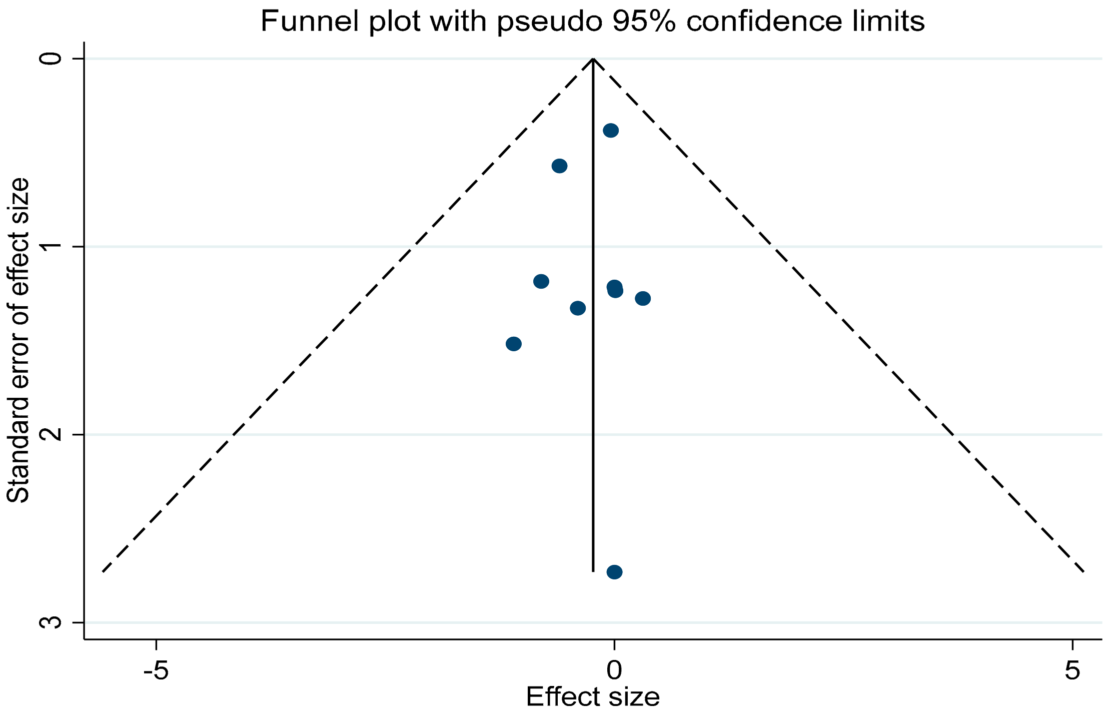

E) Fat-free mass (FFM)

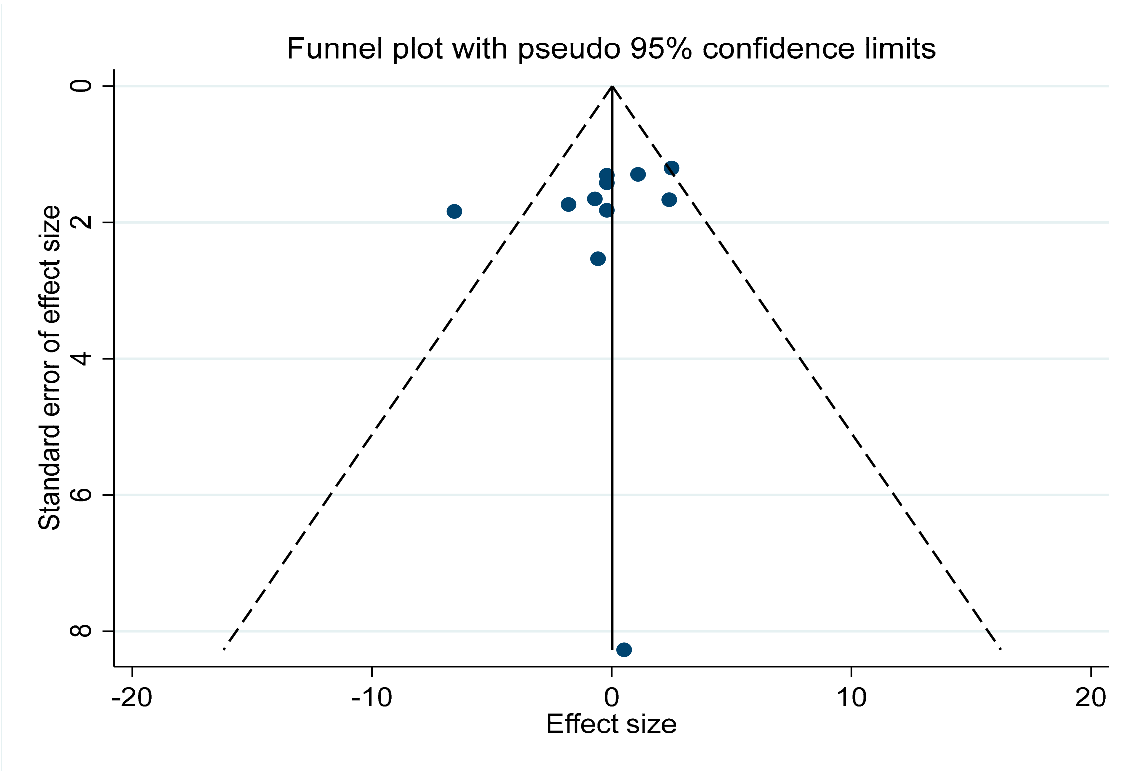

F) Waist circumference (WC)

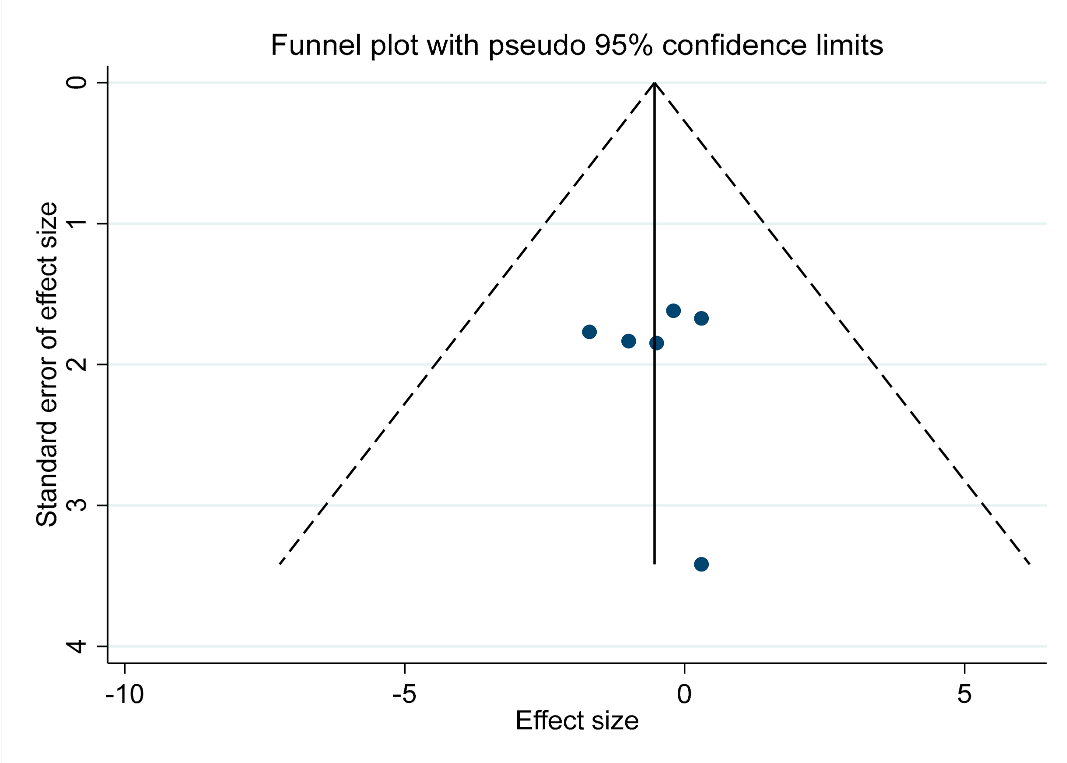

Figure S1. Funnel plots for the effects of CIT supplementation on **(A)** body weight, **(B)** body mass index, **(C)** fat mass, **(D)** body fat percentage, **(E)** fat-free mass, and **(F)** waist circumference.

A) Body weight

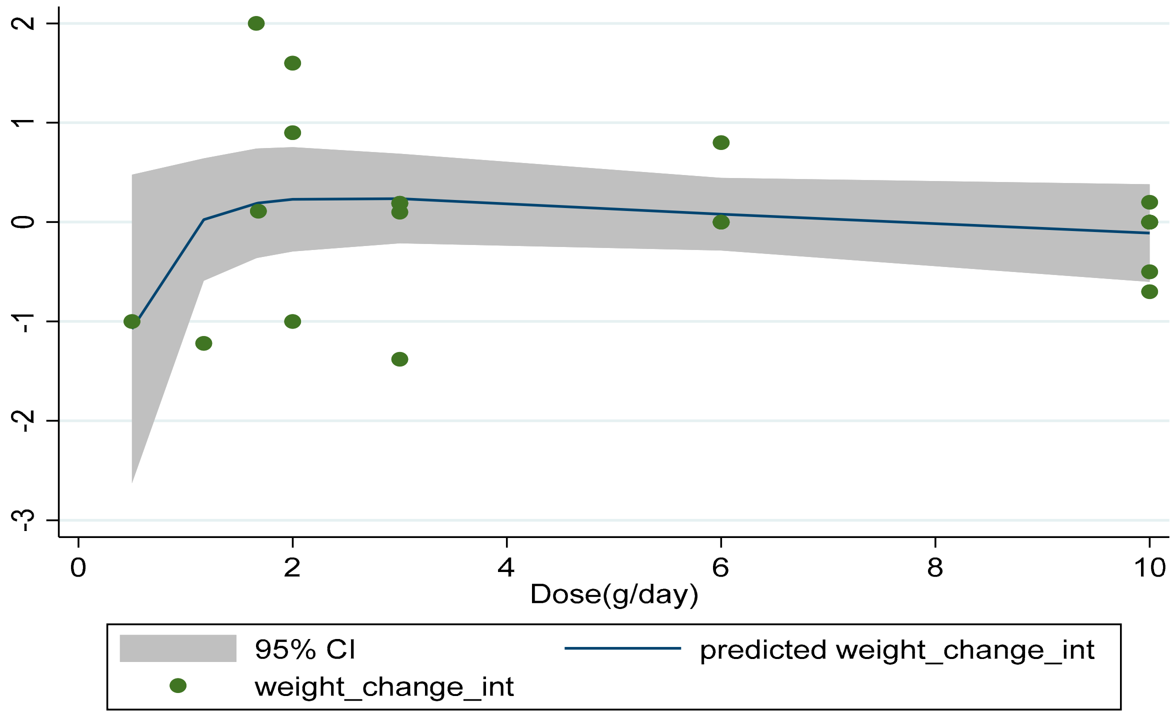

B) Body mass index (BMI)

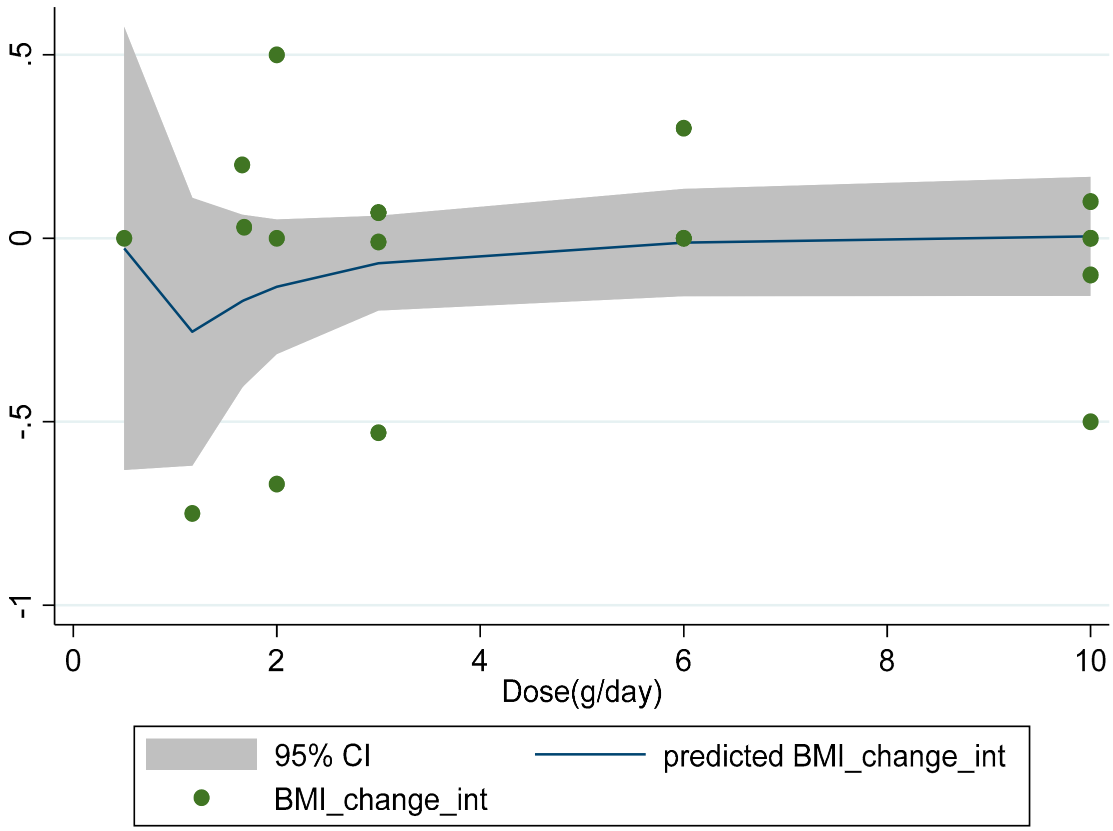

C) Fat mass (FM)

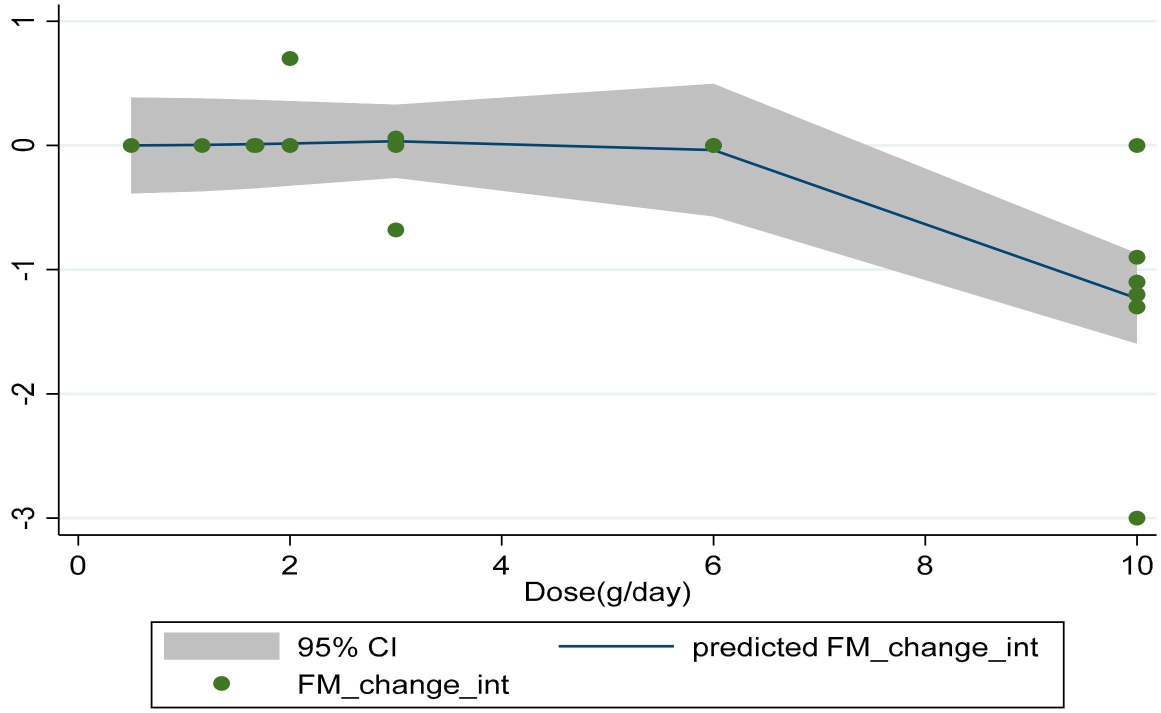

D) Body fat percentage (BFP)

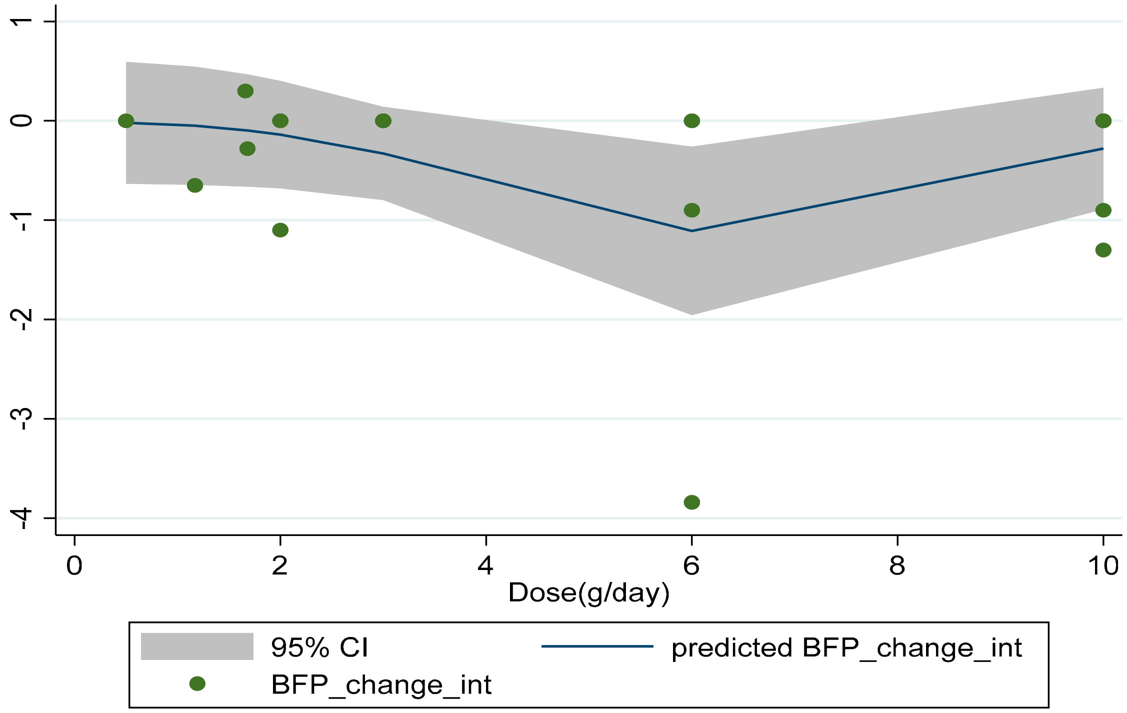

E) Fat-free mass (FFM)

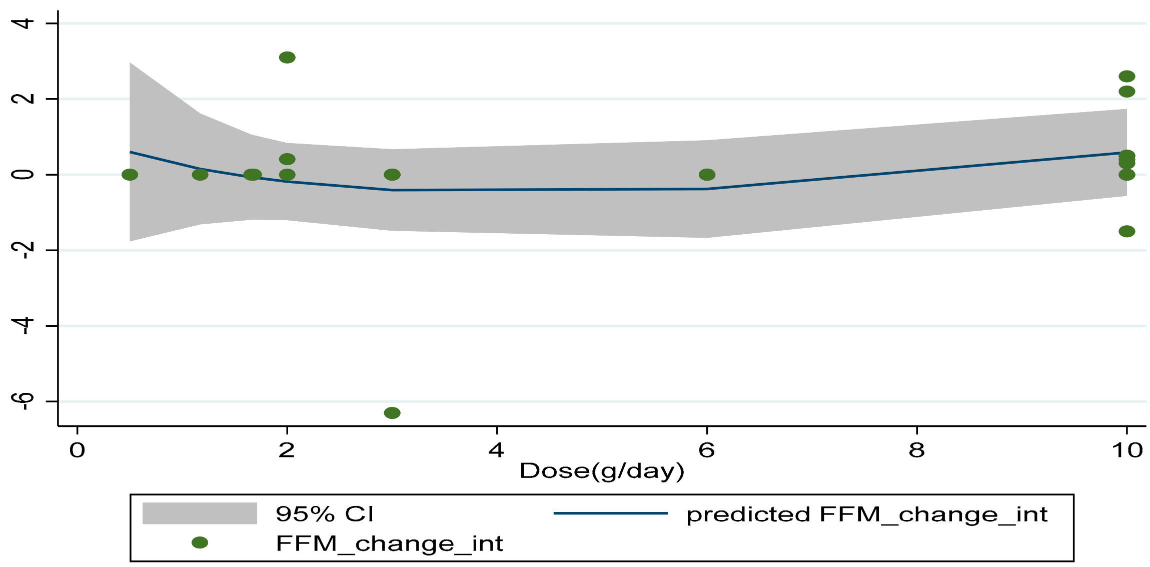

F) Waist circumference (WC)

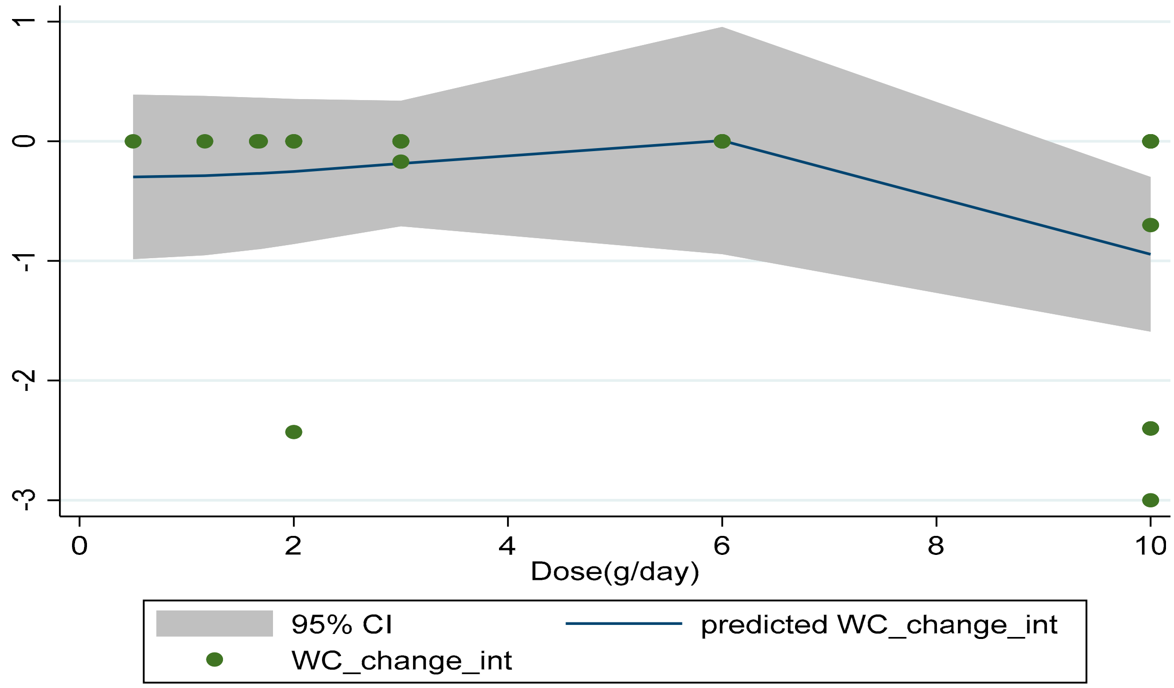

Figure S2. Non-linear dose-response association between the dose (g/day) of supplementation with CIT and absolute mean differences in **(A)** body weight (Kg), **(B)** body mass index (kg/m^2), **(C)** fat mass (kg), **(D)** body fat percentage (%), **(E)** fat-free mass (Kg), and **(F)** waist circumference (cm). The 95% CI is depicted in the shaded parts.

A) **Body weight**

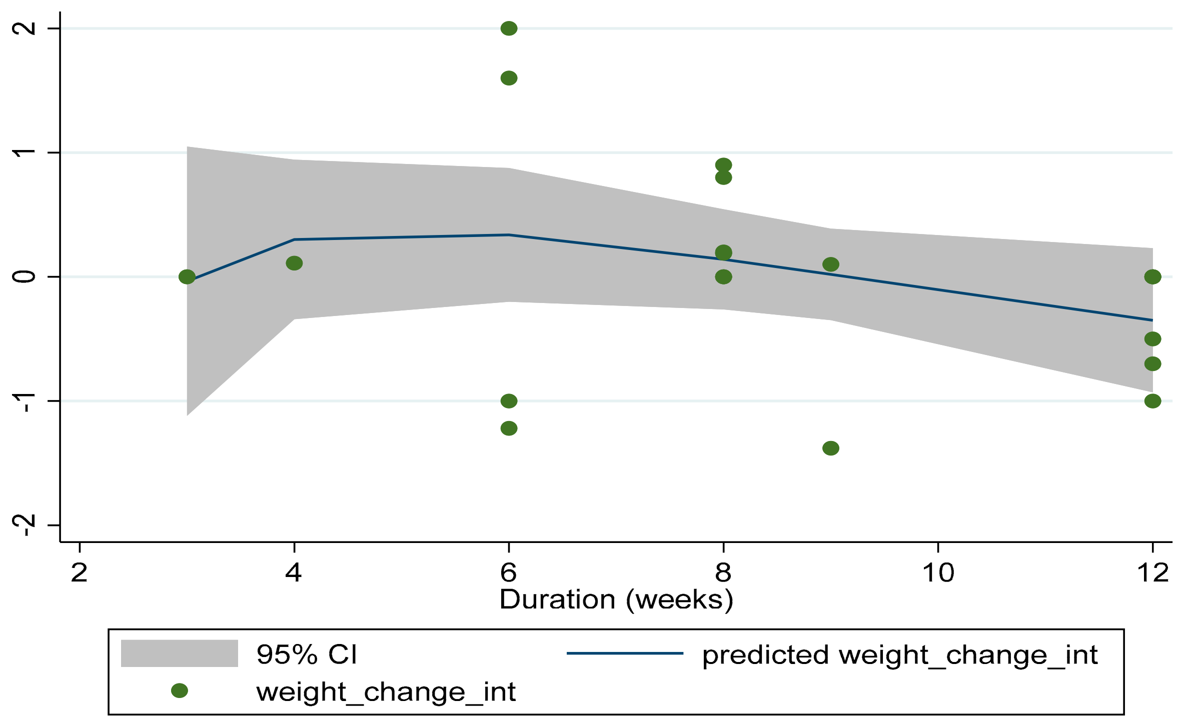

B) **Body mass index (BMI)**

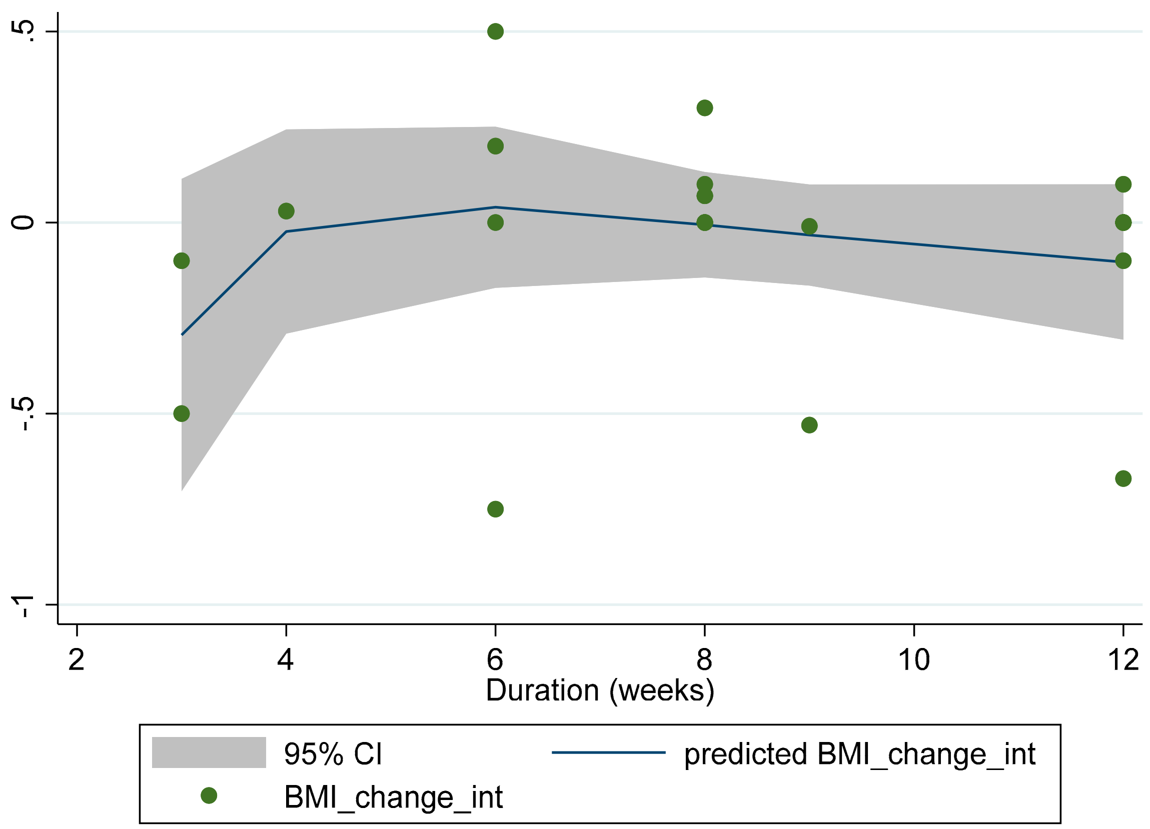

C) Fat mass (FM)

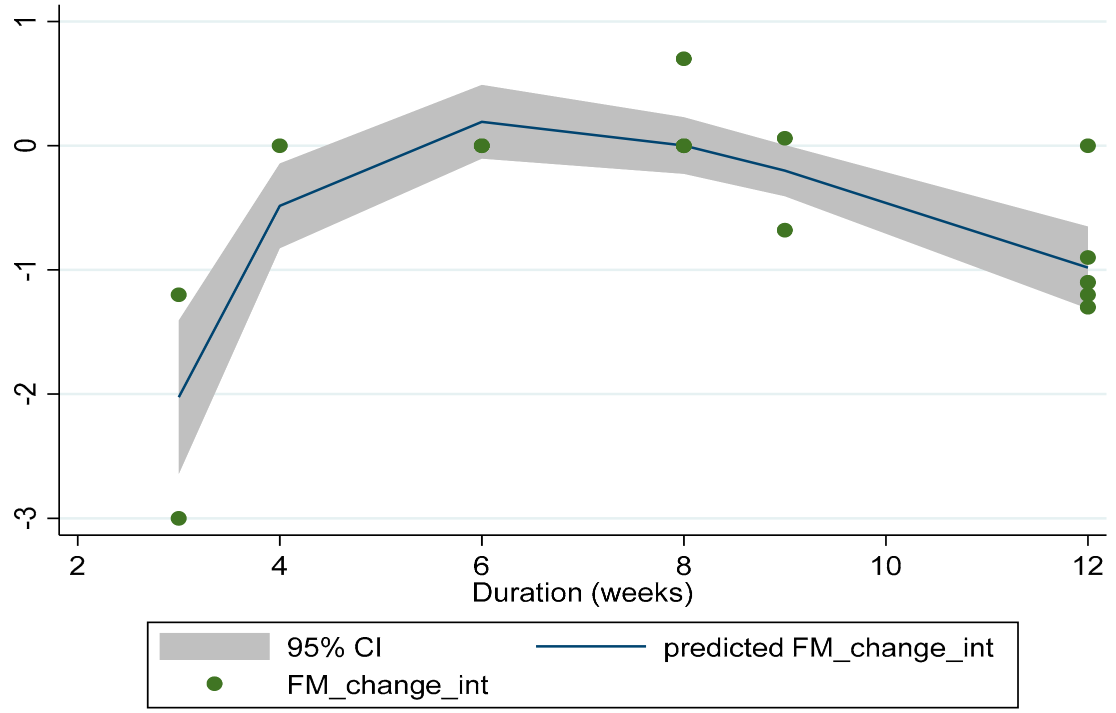

D) Body fat percentage (BFP)

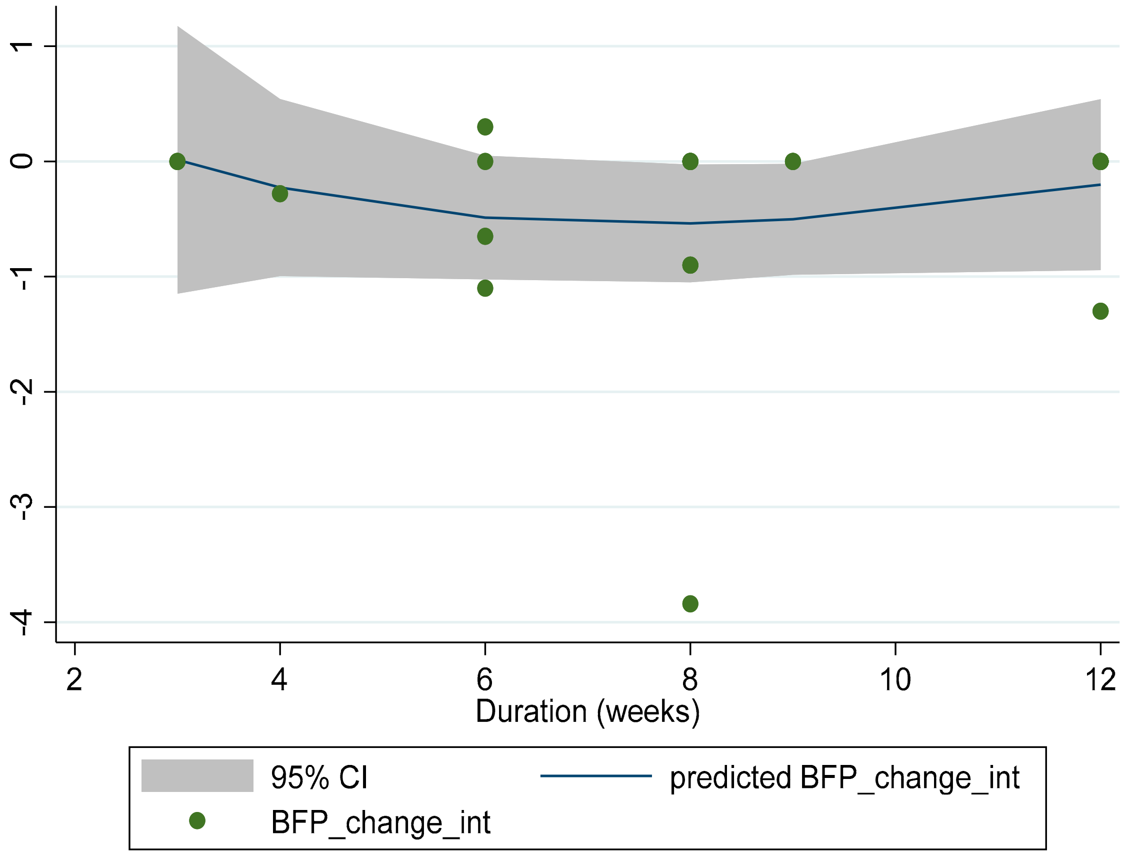

E) Fat-free mass (FFM)

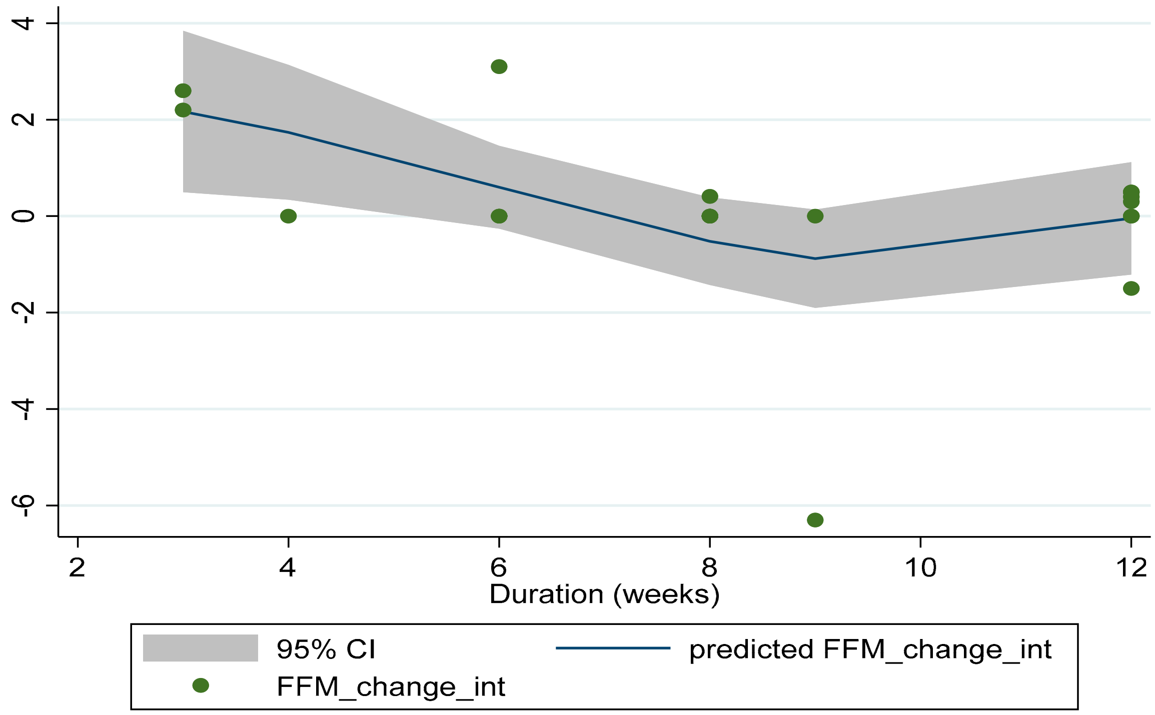

F) Waist circumference (WC)

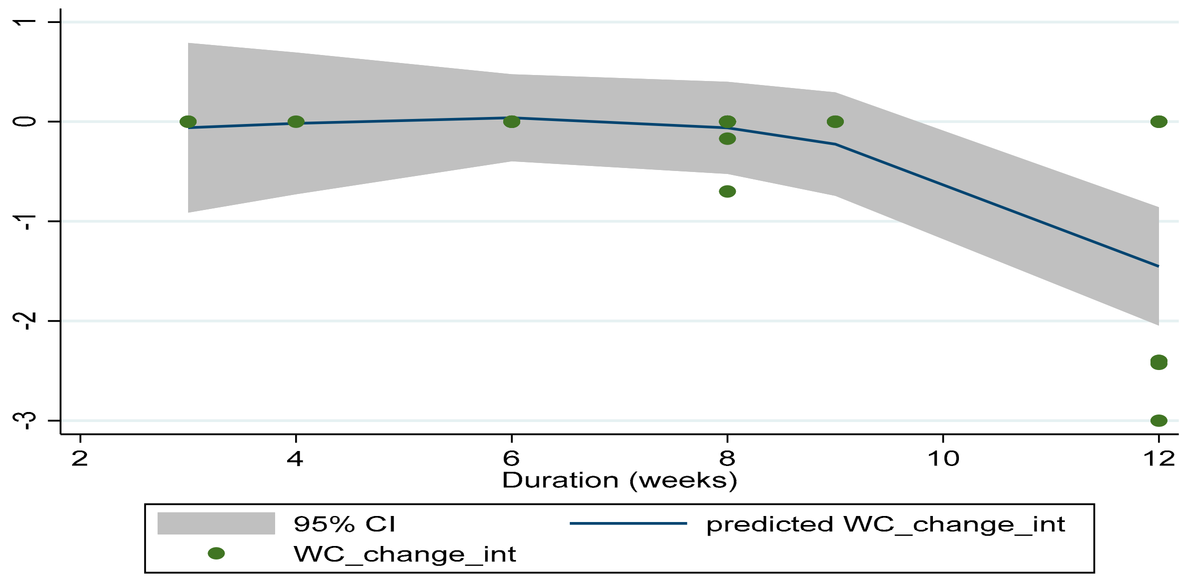

Figure S3. Non-linear dose-response association between the duration of supplementation with CIT (days) and absolute mean differences in **(A)** body weight (Kg), **(B)** body mass index (kg/m^2), **(C)** fat mass(kg), **(D)** body fat percentage (%), **(E)** fat-free mass (Kg), and **(F)** waist circumference (cm). The 95% CI is depicted in the shaded parts.

A) Body weight

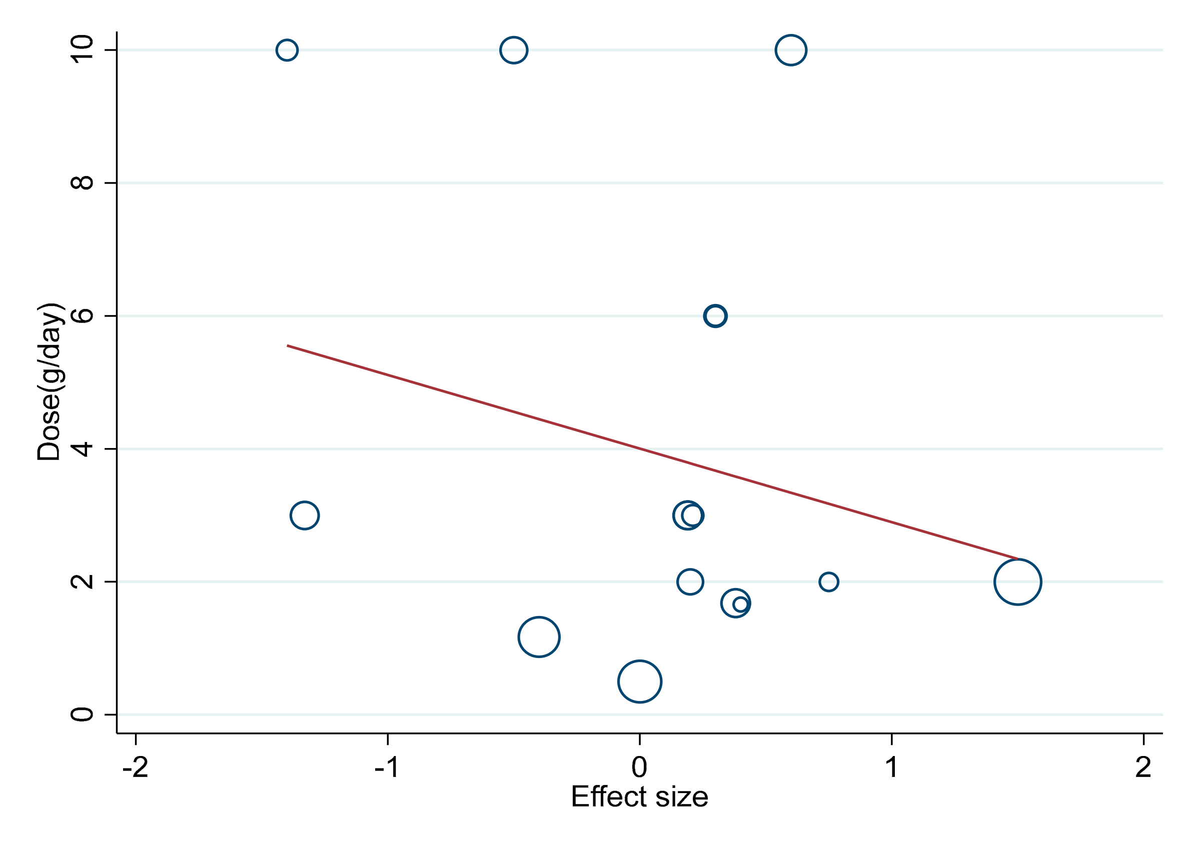

B) Body mass index (BMI)

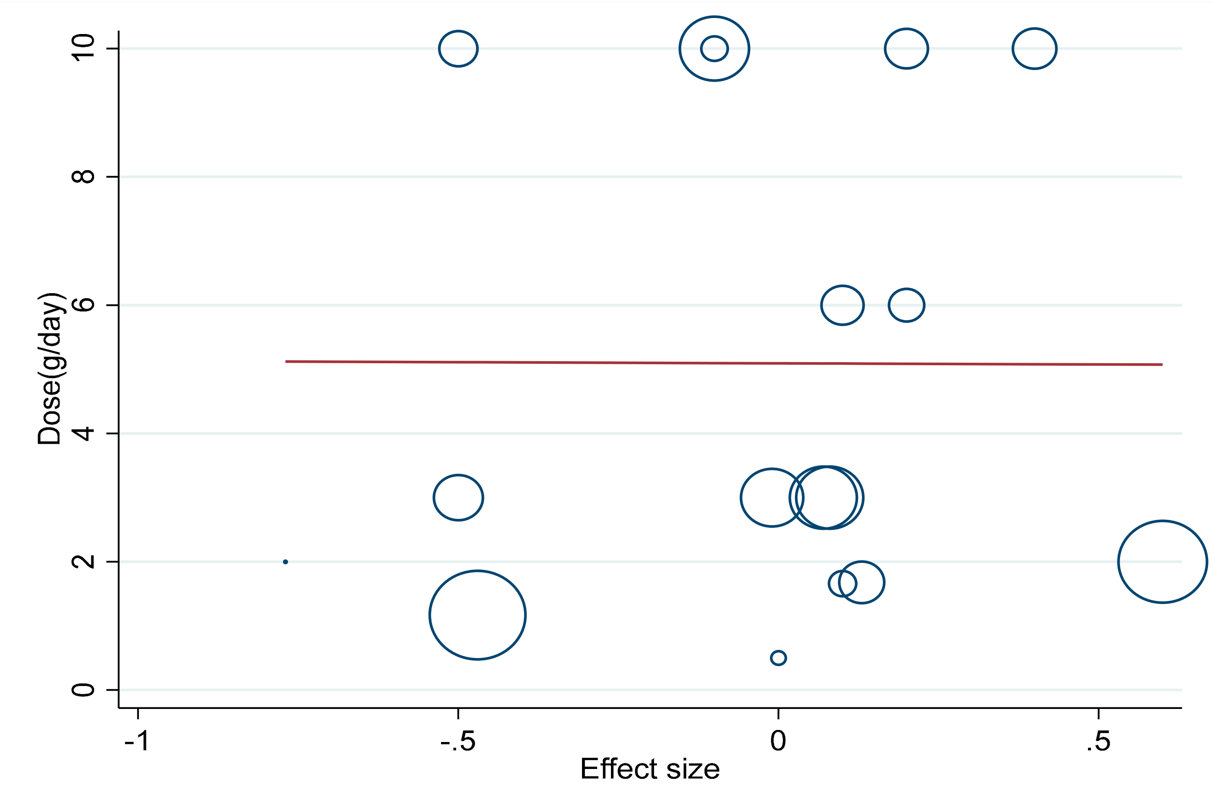

C) Fat mass (FM)

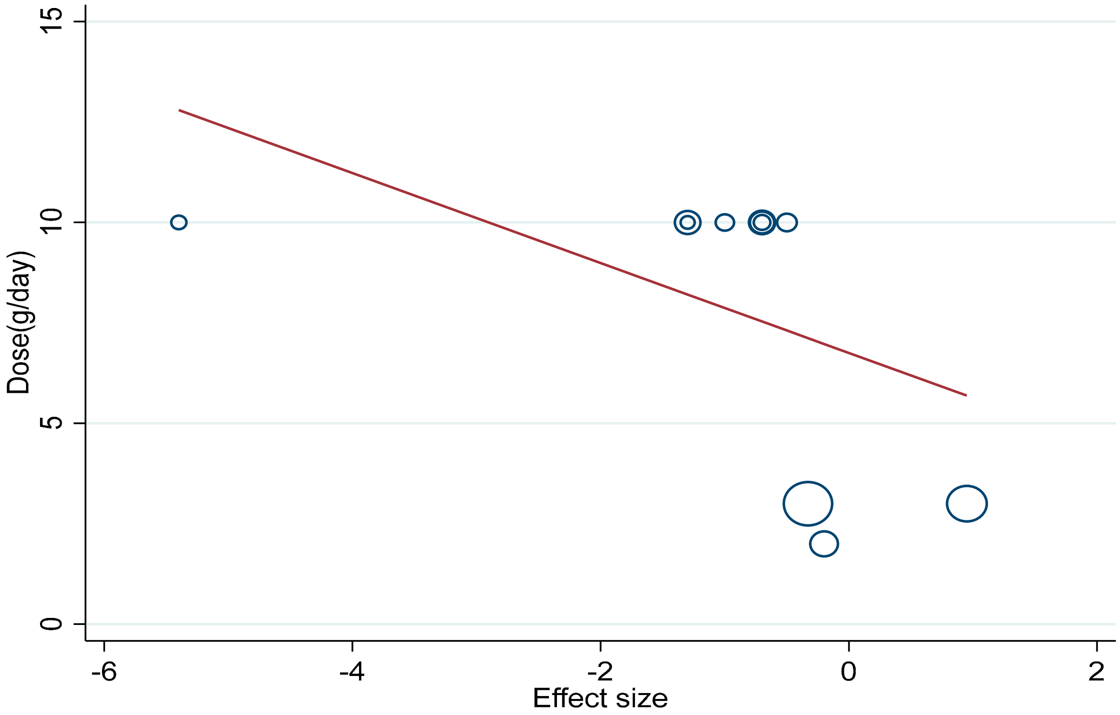

D) Body fat percentage (BFP)

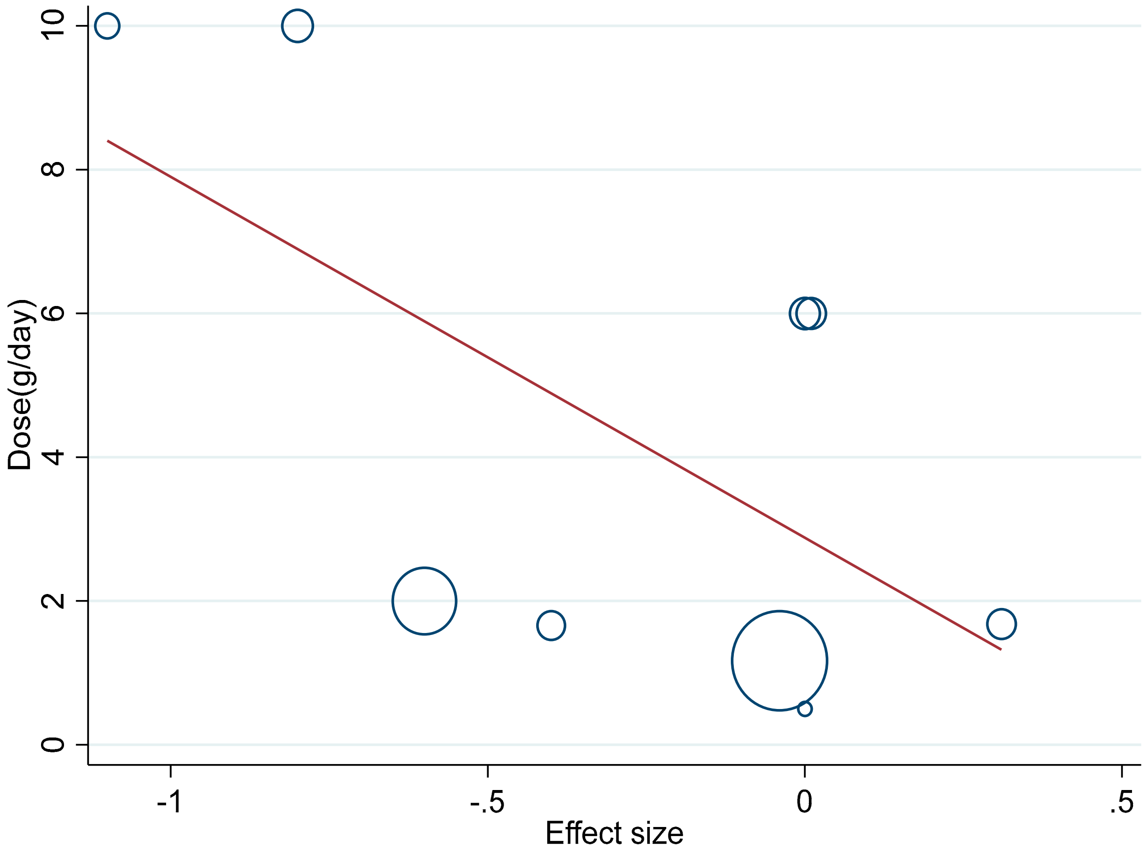

E) Fat-free mass (FFM)

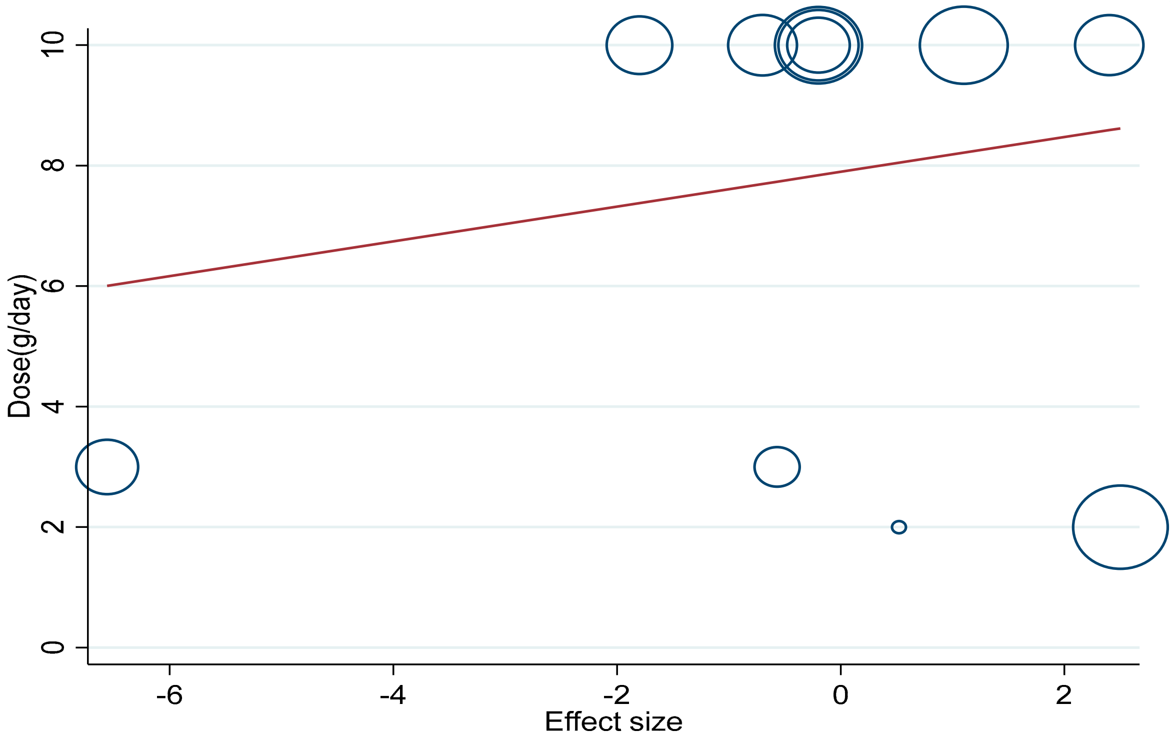

F) Waist circumference (WC)

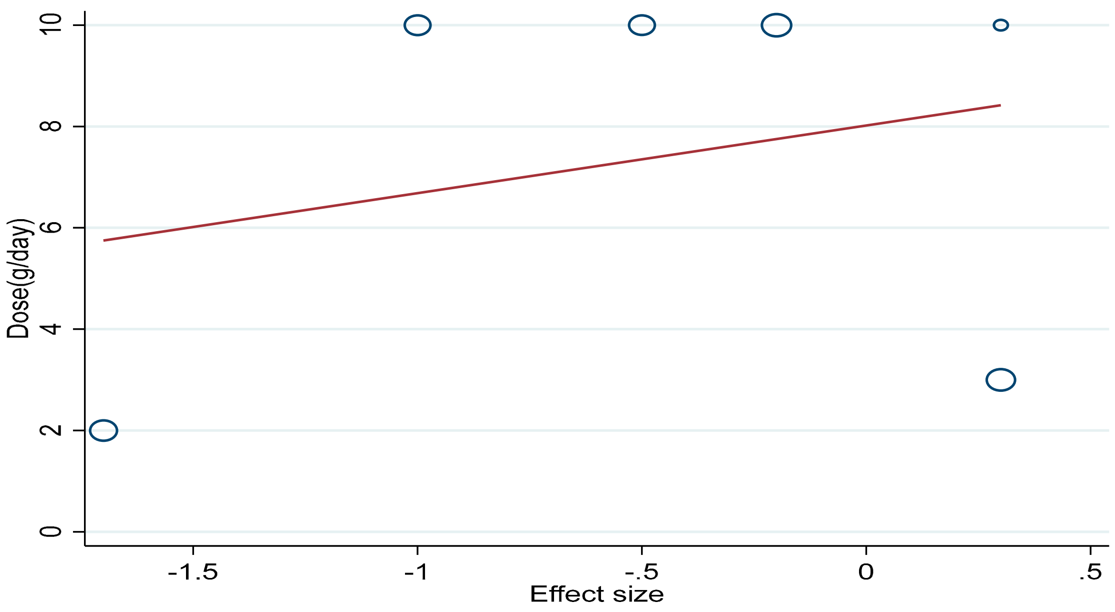

Figure S4. Linear dose-response association between dose (g/day) of CIT supplementation and absolute mean differences in **(A)** body weight (Kg), **(B)** body mass index (kg/m^2), **(C)** fat mass(kg), **(D)** body fat percentage (%), **(E)** fat-free mass (Kg), and **(F)** waist circumference (cm).

A) **Body weight**

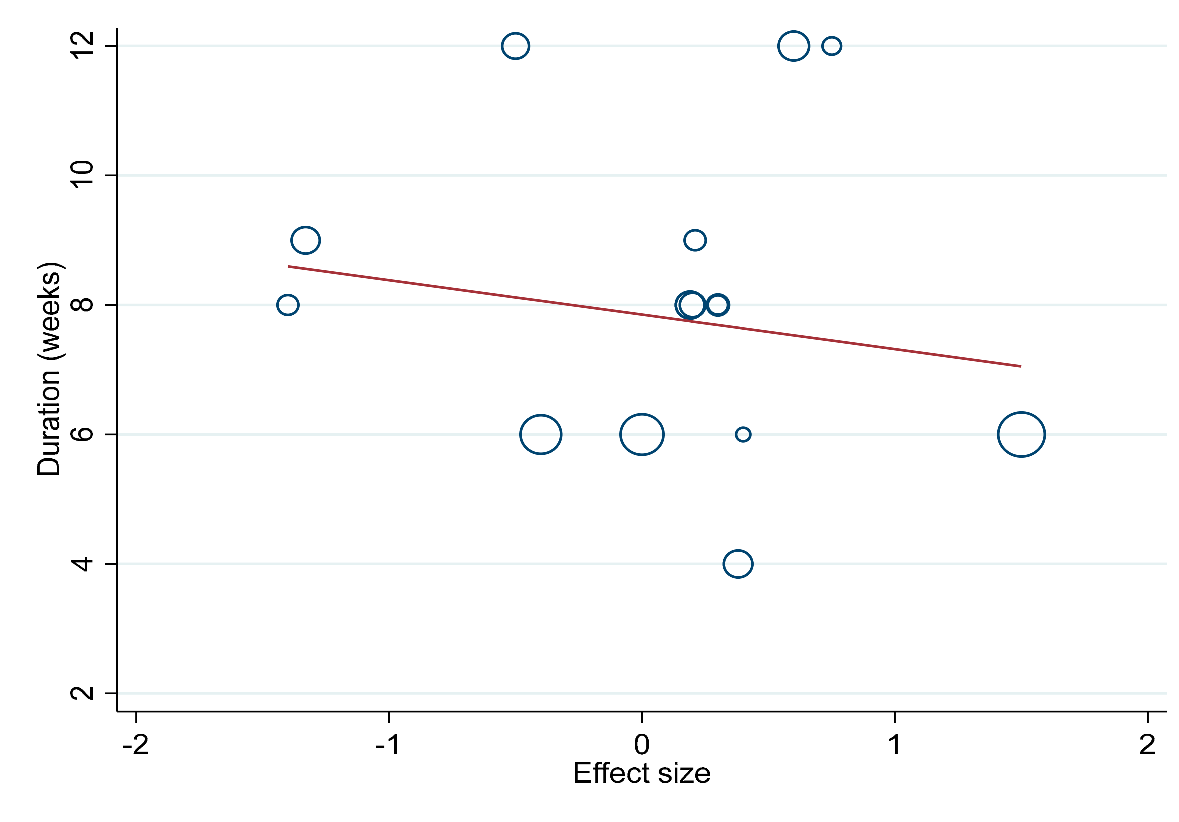

B) **Body mass index (BMI)**

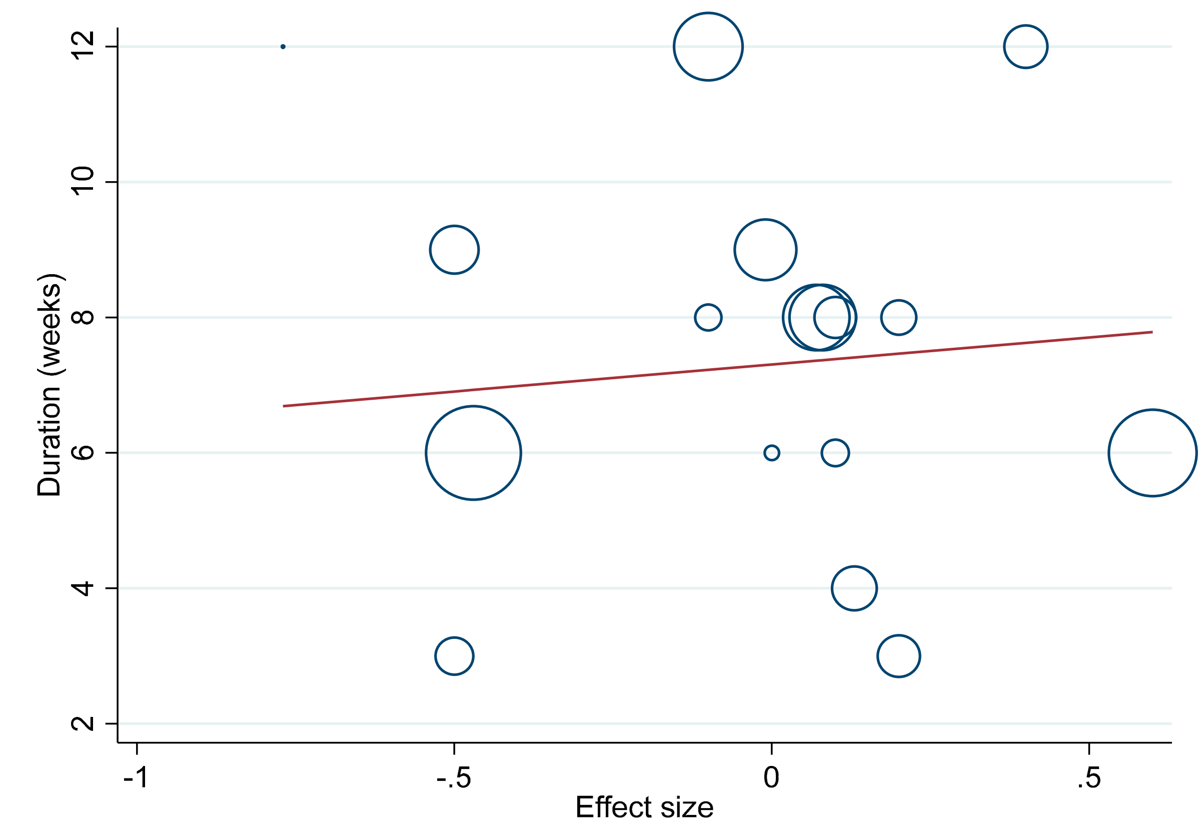

C) **Fat mass (FM)**

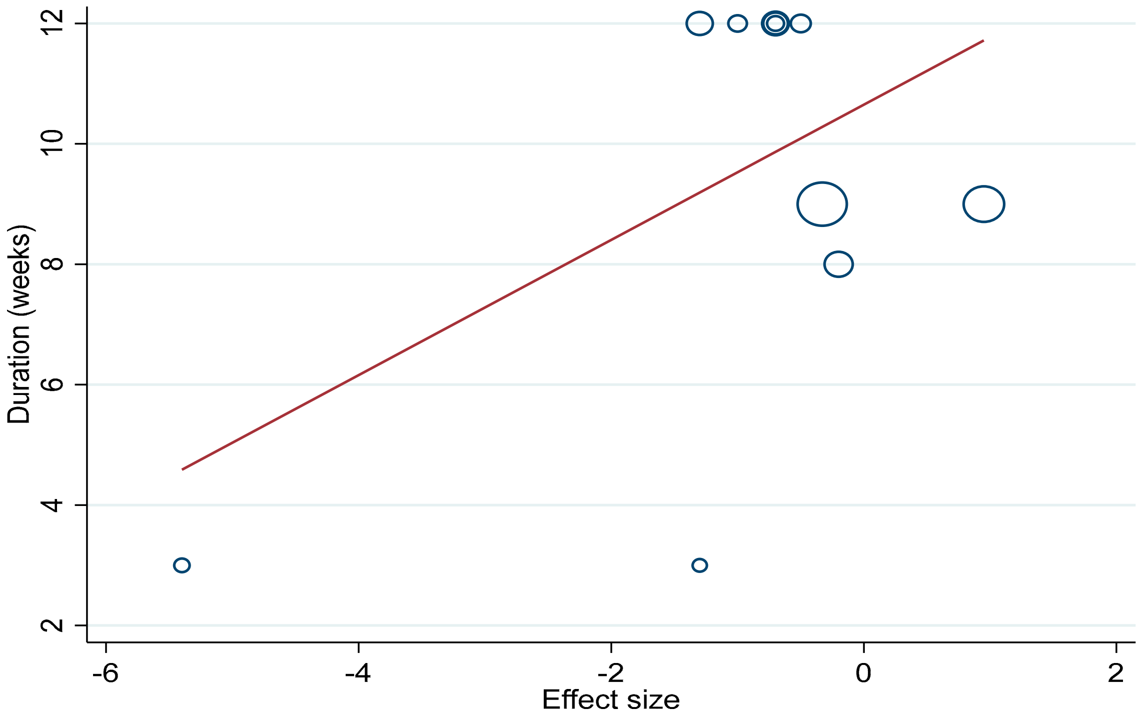

D) **Body fat percentage (BFP)**

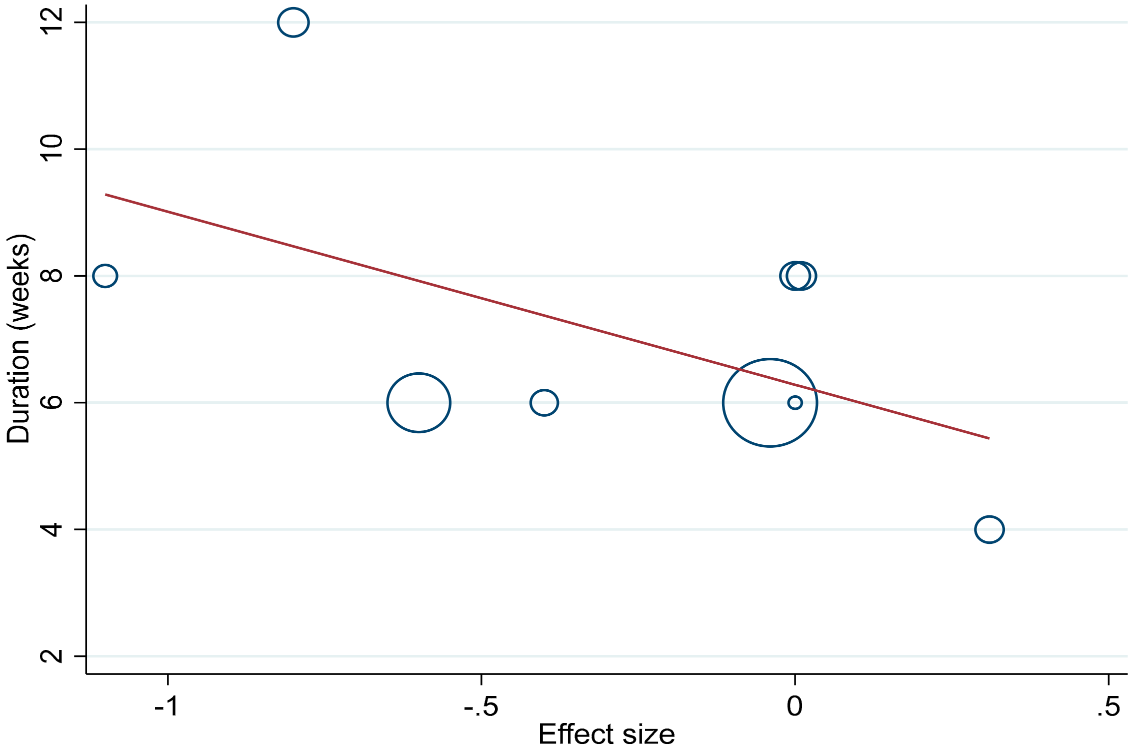

E) Fat-free mass (FFM)

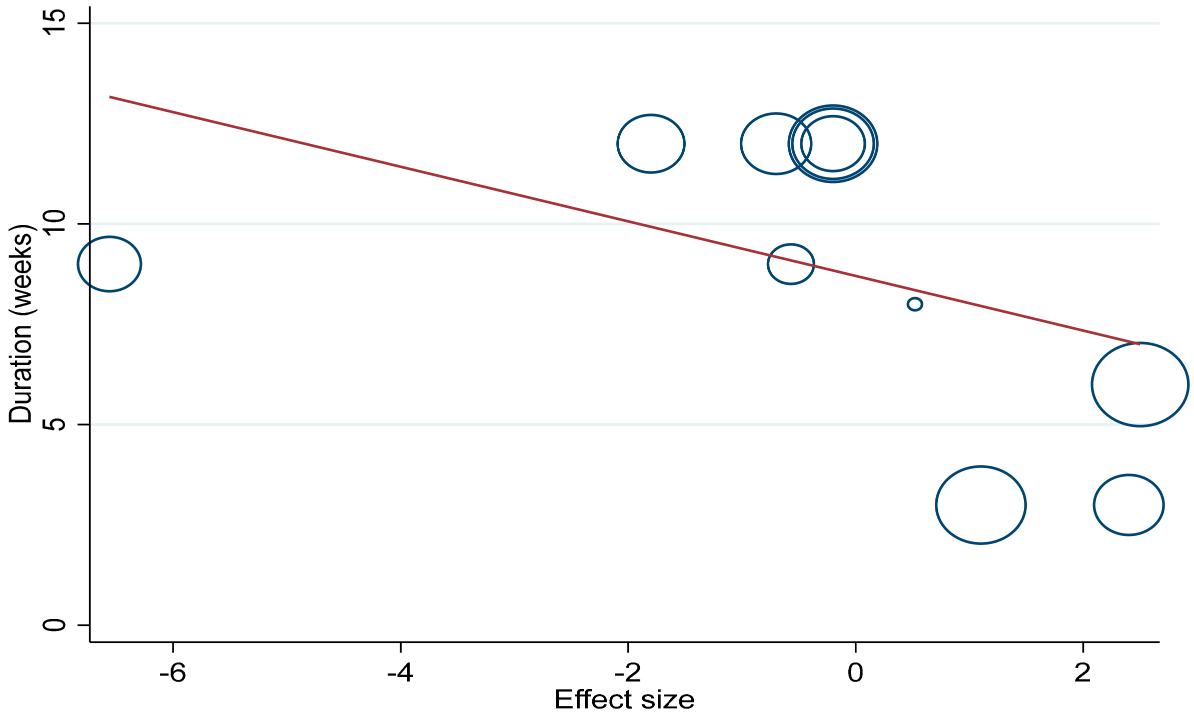

F) Waist circumference (WC)

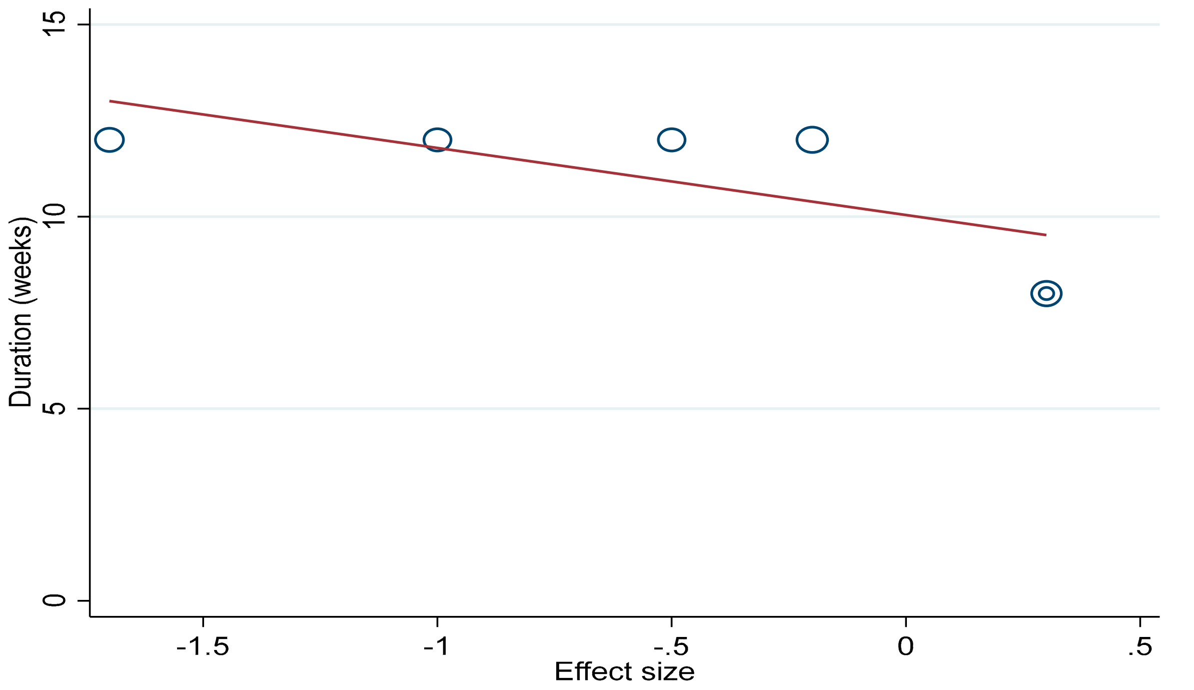

Figure S5. Linear dose-response association between the duration of supplementation with CIT (days) and absolute mean differences in **(A)** body weight (Kg), **(B)** body mass index (kg/m²), **(C)** fat mass (kg), **(D)** body fat percentage (%), **(E)** fat-free mass (Kg), and **(F)** waist circumference (cm).
